# Proline oxidase silencing induces proline-dependent pro-survival pathways in MCF-7 cells

**DOI:** 10.18632/oncotarget.24466

**Published:** 2018-02-09

**Authors:** Ilona Zareba, Katarzyna Celinska-Janowicz, Arkadiusz Surazynski, Wojciech Miltyk, Jerzy Palka

**Affiliations:** ^1^ Department of Medicinal Chemistry, Medical University of Bialystok, 15-222 Bialystok, Poland; ^2^ Department of Pharmaceutical Analysis, Medical University of Bialystok, 15-222 Bialystok, Poland

**Keywords:** MCF-7 breast cancer cells, proline dehydrogenase/proline oxidase, apoptosis, autophagy, proline

## Abstract

Proline degradation by proline dehydrogenase/proline oxidase (PRODH/POX) contributes to apoptosis or autophagy. The identification of specific pathway of apoptosis/survival regulation is the aim of this study. We generated knocked-down PRODH/POX MCF-7 breast cancer cells (MCF-7^shPRODH/POX^). PRODH/POX silencing did not affect cell viability. However, it contributed to decrease in DNA and collagen biosynthesis, increase in prolidase activity and intracellular proline concentration as well as increase in the expression of iNOS, NF-κB, mTOR, HIF-1α, COX-2, AMPK, Atg7 and Beclin-1 in MCF-7^shPRODH/POX^ cells. In these cells, glycyl-proline (GlyPro, substrate for prolidase) further inhibited DNA and collagen biosynthesis, maintained high prolidase activity, intracellular concentration of proline and up-regulated HIF-1α, AMPK, Atg7 and Beclin-1, compared to GlyPro-treated MCF-7 cells. In MCF-7 cells, GlyPro increased collagen biosynthesis, concentration of proline and expression of caspase-3, cleaved caspases -3 and -9, iNOS, NF-κB, COX-2 and AMPKβ. PRODH/POX knock-down contributed to pro-survival autophagy pathways in MCF-7 cells and GlyPro-derived proline augmented this process. However, GlyPro induced apoptosis in PRODH/POX-expressing MCF-7 cells as detected by up-regulation of active caspases -3 and -9. The data suggest that PRODH/POX silencing induces autophagy in MCF-7 cells and GlyPro-derived proline supports this process.

## INTRODUCTION

Proline oxidase (POX), known also as proline dehydrogenase (PRODH) is flavin-dependent enzyme associated with the inner mitochondrial membrane [1, 2]. The enzyme catalyzes the conversion of proline into Δ1-pyrroline-5-carboxylate (P5C). This reaction is important in maintaining the redox balance in the cells, providing mechanism for utilization of reducing potential of free proline. Alternatively, free proline is utilized in collagen biosynthesis [3, 4]. The intensity of collagen biosynthesis determines availability of free proline for PRODH/POX-dependent utilization. It seems that cytoplasmic proline that enters mitochondria is sensor of cellular energy status. During conversion of proline to P5C, electrons are transported to electron transport chain producing ATP or they directly reduce oxygen, producing reactive oxygen species (ROS). In the first situation, PRODH/POX activation produces ATP for energy supply and survival [1, 5–7]. In the second one, ROS induce apoptotic pathways [7–10].

Apoptosis can operate extrinsically, via binding of a death ligand to a death receptor on cell surface, or intrinsically. The intrinsic pathway of apoptosis is initiated by intracellular stress such as mitochondrial dysfunction that leads to the permeabilization of the mitochondria, release of apoptogenic factors from the mitochondrial intermembrane space, and subsequent execution of apoptosis [11].

Autophagy is a process in which a cell maintains plasma membrane integrity, while degrades and recycles its own intracellular components. The process is triggered by cellular stresses as oxidative stress, hypoxia, nutrient or growth factor deprivation and others. The process involves formation of autophagosomes, double-membraned vesicles, which fuse to lysosomes to form autolysosomes. Cytosolic materials previously taken up by autophagosomes are degraded by lysosomal enzymes leading to autophagic cell death [11].

In this study, we hypothesized that critical factor in PRODH/POX-dependent regulation of apoptosis/autophagy is both PRODH/POX activity and proline availability. Several studies showed increase in proline content in neoplastic cells [12, 13]. The mechanism of proline accumulation in neoplastic cells is not fully understood. The main pool of intracellular proline comes from extracellular collagen degradation [14]. The process is initiated by extracellular metalloproteinases and then collagen degradation products are further processed intracellularly in lysosomes to free amino acids, except imidodipeptides, e.g. glycyl-proline. Imidodipeptides are cleaved to amino acids in cytoplasm by Prolidase [E.C.3.4.13.9]. This enzyme plays important role in regulation of proline-dependent metabolic responses [15, 16]. It seems that important role in regulation of proline level in cytoplasm plays also conversion of P5C to proline through P5C reductase (PYCR), the NADPH/NADH-dependent enzyme. This pathway was found to be coupled to pentose phosphate pathway and glucose metabolism [1, 2, 17, 18].

Proline plays also important role as an inhibitor of degradation of hypoxia inducible factor (HIF-1α), transcription factor that activates several “pro-survival” genes, as vascular endothelial growth factor (VEGF), cyclo-oxygenase-2 (COX-2) and nuclear factor κB (NF-κB) [15, 19, 20]. The accumulation of proline or its utilization may represent important switch in the regulation of HIF-dependent functions [21].

The role of PRODH/POX in regulation of breast cancer cell apoptosis/autophagy is not known. One of the best characterized breast cancer cells in respect to collagen biosynthesis, prolidase activity and PRODH/POX expression is MCF-7 cell line. The cells express both enzymes and synthesize collagen [22–24]. Therefore, we used MCF-7^shPRODH/POX^ cell line as a model to study mechanisms underlying PRODH/POX-dependent pro-apoptotic/pro-survival pathways in differential conditions of proline availability for degradation by PRODH/POX. The rational to use MCF-7 cells was also that in contrast to other breast cancer cells, e.g. MDA-MB-231, they evoke relatively high expression of PRODH/POX. It was also of great importance that MCF-7 cells express both α and β estrogen receptors while MDA-MB-231 cells are lacking α-estrogen receptors. Previously we have found that the rate of collagen biosynthesis in breast cancer cells is dependent on the status of α estrogen receptor [22].

Elegant studies (cited in this paper) of James Phang’s group documented PRODH/POX as a mitochondrial tumor suppressor. The mechanism of PRODH/POX-dependent inhibition of cancer cell proliferation may undergo through modulation of cell signaling pathways. PRODH/POX was shown to modulate epidermal growth factor receptor (EGFR) and mitogen-activated protein kinase (MAPK) signaling, down-regulate COX-2 expression and Wnt/β-catenin pathway [25–27]. These processes are likely to be related to PRODH/POX-dependent ROS signaling and subsequent activation of caspases or regulation by transcription factors. MCF-7 cells express wild-type p53 protein [28]. PRODH/POX expression is regulated by P53 [29–31], while PRODH/POX down regulates HIF-1α signaling [32], that mediate glycolysis, angiogenesis, metastasis and survival [15]. It seems that PRODH/POX-dependent inhibition of HIF-1α could play important role in inhibition of cancer cell growth and invasion. However, in certain environmental conditions like stress situations (oxygen or glucose deficiency, inflammation) PRODH/POX may act as a pro-survival factor [5, 33–36] inducing expression of AMPK for protective autophagy [35].

Based on the above findings, the identification of PRODH/POX-dependent pathway that potentially is involved in regulation of apoptosis/survival is of special interest. Therefore, we evaluated the effect of PRODH/POX silencing on several above mention factors affecting apoptosis and survival in MCF-7 cells.

## RESULTS

To study the effect of PRODH/POX silencing on metabolism in MCF-7 cells we designed 3 different constructs of shRNA for PRODH/POX and prepared 3 clones of PRODH/POX knocked-down MCF-7 cells, respectively (Supplementary Material, Supplementary Figure 1).

We studied the effect of PRODH/POX knock-down on cell viability, DNA and collagen biosynthesis, prolidase activity and intracellular proline concentration (Supplementary Material, Supplementary Figure 2). We found that clone 2 MCF-7 cells represent the most characteristic phenotype to study the role of deregulation of proline generation/utilization processes on apoptosis/autophagy. Therefore, we used clone 2 MCF-7 cells (MCF-7^shPRODH/POX^) for further studies.

### Effect of PRODH/POX silencing and glycyl-proline (GlyPro) on cell viability, DNA biosynthesis, collagen biosynthesis, prolidase activity and intracellular proline concentration in MCF-7 cells

**Figure 1:**
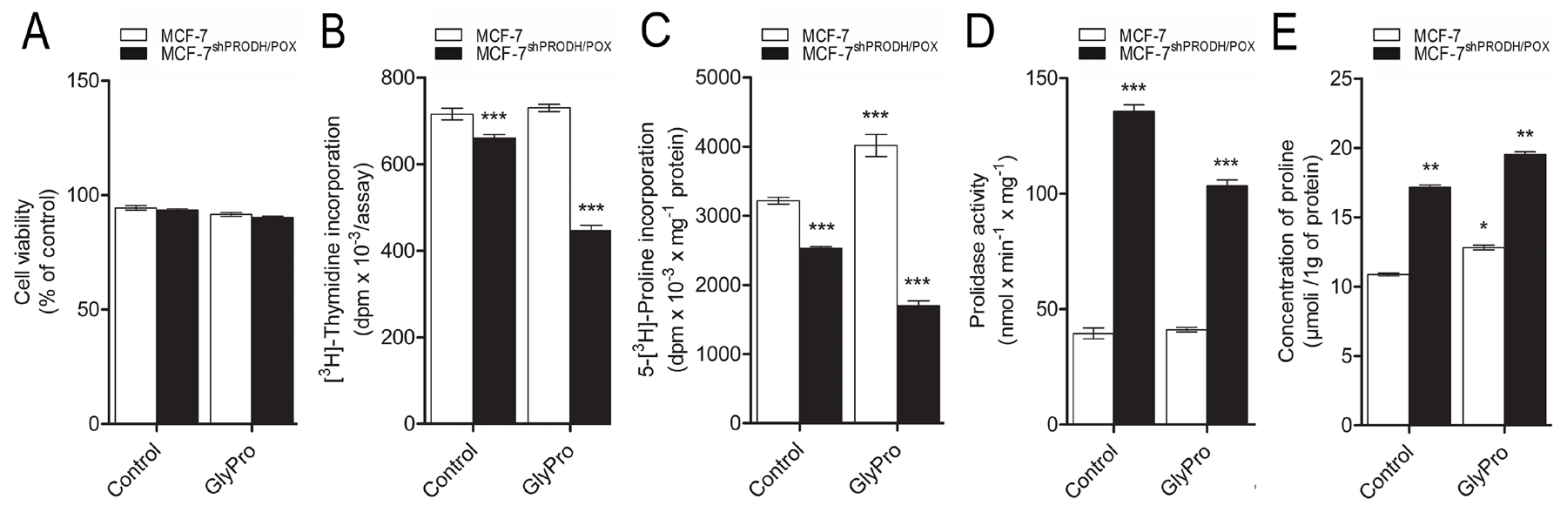
Effect of PRODH/POX silencing and GlyPro on cell viability **(A)**, DNA biosynthesis **(B)**, collagen biosynthesis **(C)**, prolidase activity **(D)** and intracellular proline concentration **(E)** in MCF-7 and MCF-7shPRODH/POX cells. The mean values ± SEM from 3 experiments done in duplicates are presented. Asterisks indicate differences between control MCF-7 cells and MCF-7^shPRODH/POX^ cells at ^*^P<0.05, ^**^P<0.01 and ^***^P<0.001.

The studies were performed on PRODH/POX silenced MCF-7 cells (MCF-7^shPRODH/POX^) and MCF-7 control cells. Considering important role of proline in the above processes, we used GlyPro as a substrate for prolidase in order to increase cytoplasmic level of proline. There was no significant difference in cell viability between MCF-7^shPRODH/POX^ and MCF-7 control cells, as well as the cells treated with GlyPro (Figure 1A). However, in MCF-7^shPRODH/POX^ cells DNA biosynthesis was significantly decreased compared to MCF-7 cells (Figure 1B). Although, there was not significant effect of GlyPro on DNA biosynthesis in MCF-7 cells, it further inhibited the process in MCF-7^shPRODH/POX^ cells.

Collagen biosynthesis was decreased in MCF-7^shPRODH/POX^ cells, compared to MCF-7 cells and GlyPro further inhibited the process (Figure 1C). However, significant increase in collagen biosynthesis was found in GlyPro treated MCF-7 cells.

PRODH/POX silencing significantly increased prolidase activity in both GlyPro treated and untreated MCF-7 cells, compared to control cells (Figure 1D). There was not significant difference in prolidase activity in GlyPro treated MCF-7 cells.

Concentration of intracellular proline was increased in MCF-7^shPRODH/POX^ compared to MCF-7 control cells, as well as in both cell lines treated with GlyPro (Figure 1E).

### Effect of PRODH/POX silencing and GlyPro on pro-apoptotic and pro-survival pathways in MCF-7 cells

**Figure 2:**
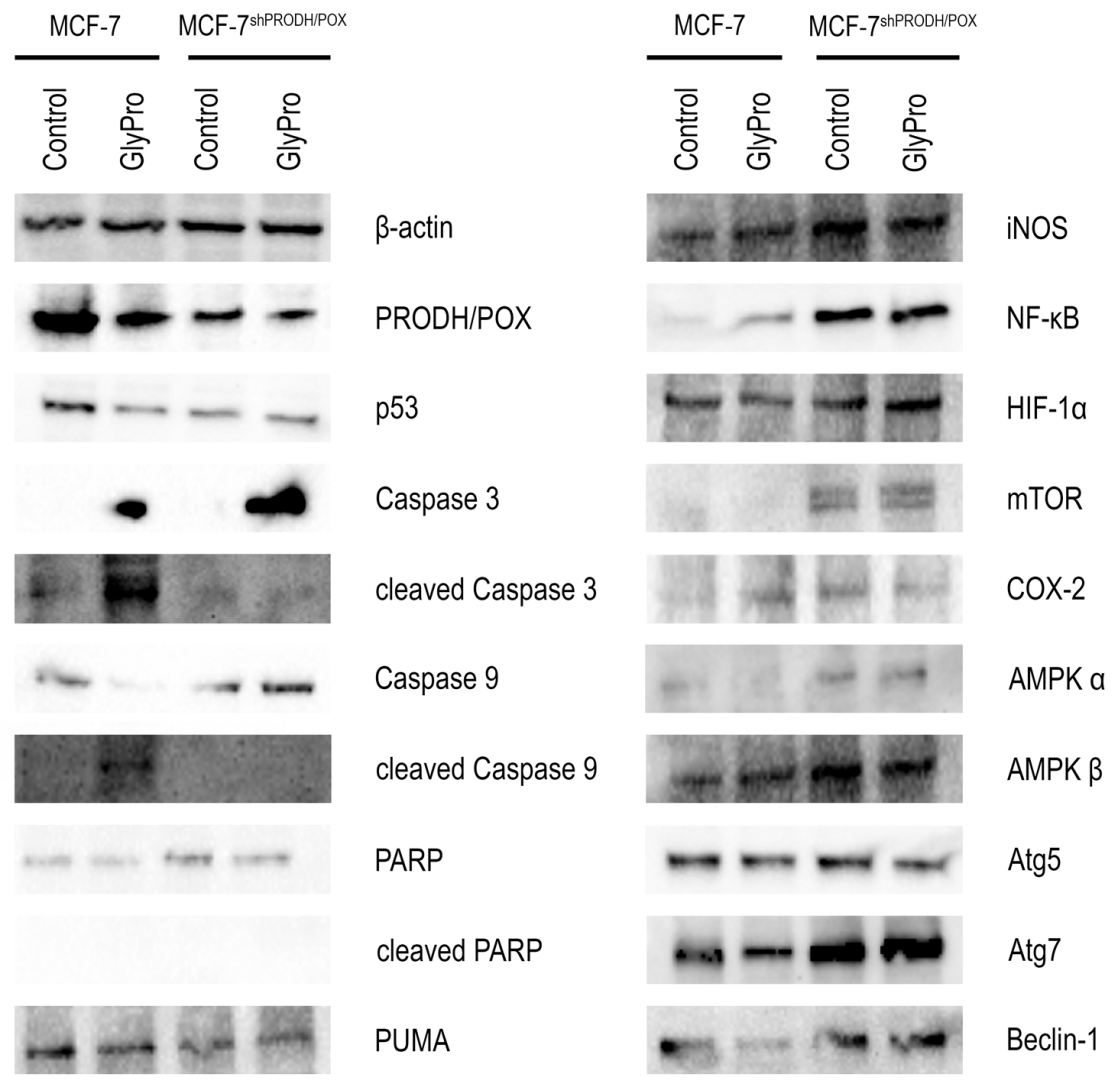
Effect of PRODH/POX silencing and GlyPro on pro-apoptotic and pro-autophagy signaling in MCF-7 cells Western blot analysis for PRODH/POX, p53, Caspase 3, cleaved-Caspase 3, Caspase-9, cleaved-Caspase 9, PARP, cleaved-PARP, PUMA, iNOS, NF-κB, HIF-1α, mTOR, COX-2, AMPKα, AMPKβ, Atg5, Atg7 and Beclin-1 in MCF-7 and MCF-7^shPRODH/POX^ cells and the cells treated with GlyPro. Representative blots obtained from 3 experiments done in duplicates are presented. Samples used for electrophoresis consisted of 20 μg protein of cells homogenate extracts.

To test the role of PRODH/POX silencing in apoptosis-inducing pathways we analyzed expression of PRODH/POX, p53, Caspase-3, Caspase-9, PARP and PUMA in GlyPro treated and untreated MCF-7 and MCF-7^shPRODH/POX^ cells (Figure 2, Supplementary Figure 3). Expressions of PRODH/POX and p53 were decreased in MCF-7^shPRODH/POX^ cells compared to MCF-7 cells. GlyPro-treatment decreased expression of both proteins only in MCF-7 cells. The expression of non-active Caspase-3 was found in both cell lines treated with GlyPro, while the high expression of cleaved form of Caspase 3 was detected only in GlyPro-treated MCF-7 cells. In respect to non-active Caspase 9, the expression was similar in both cell lines, however, in MCF-7 cells treated with GlyPro the expression was decreased while in MCF-7^shPRODH/POX^ cells it was stimulated. Cleaved form of Caspase 9 was expressed only in MCF-7 cells treated with GlyPro. Expression of non-active PARP was more pronounced in MCF-7^shPRODH/POX^ cells than in MCF-7 cells and GlyPro had no effect on the process. However, there was no expression of active PARP in both cell lines (treated and untreated with GlyPro). The expression of PUMA was more pronounced in MCF-7 cells than in MCF-7^shPRODH/POX^ cells and GlyPro had no effect on the process.

To test the role of PRODH/POX silencing in pro-survival pathways we analyzed expression of iNOS, NFκB, HIF-1α, mTOR, COX-2, Atg5, Atg7, Beclin-1 and AMPKs in control and GlyPro treated MCF-7 and MCF-7^shPRODH/POX^ cells (Figure 2, Supplementary Figure 3). Expression of iNOS, NFκB, HIF-1α, mTOR, COX-2, AMPKα, AMPKβ, Atg7 and Beclin-1 were increased in both GlyPro treated and untreated MCF-7^shPRODH/POX^ cells compared to MCF-7 cells. There was no difference in expression of Atg5 in both cell lines.

**Figure 3:**
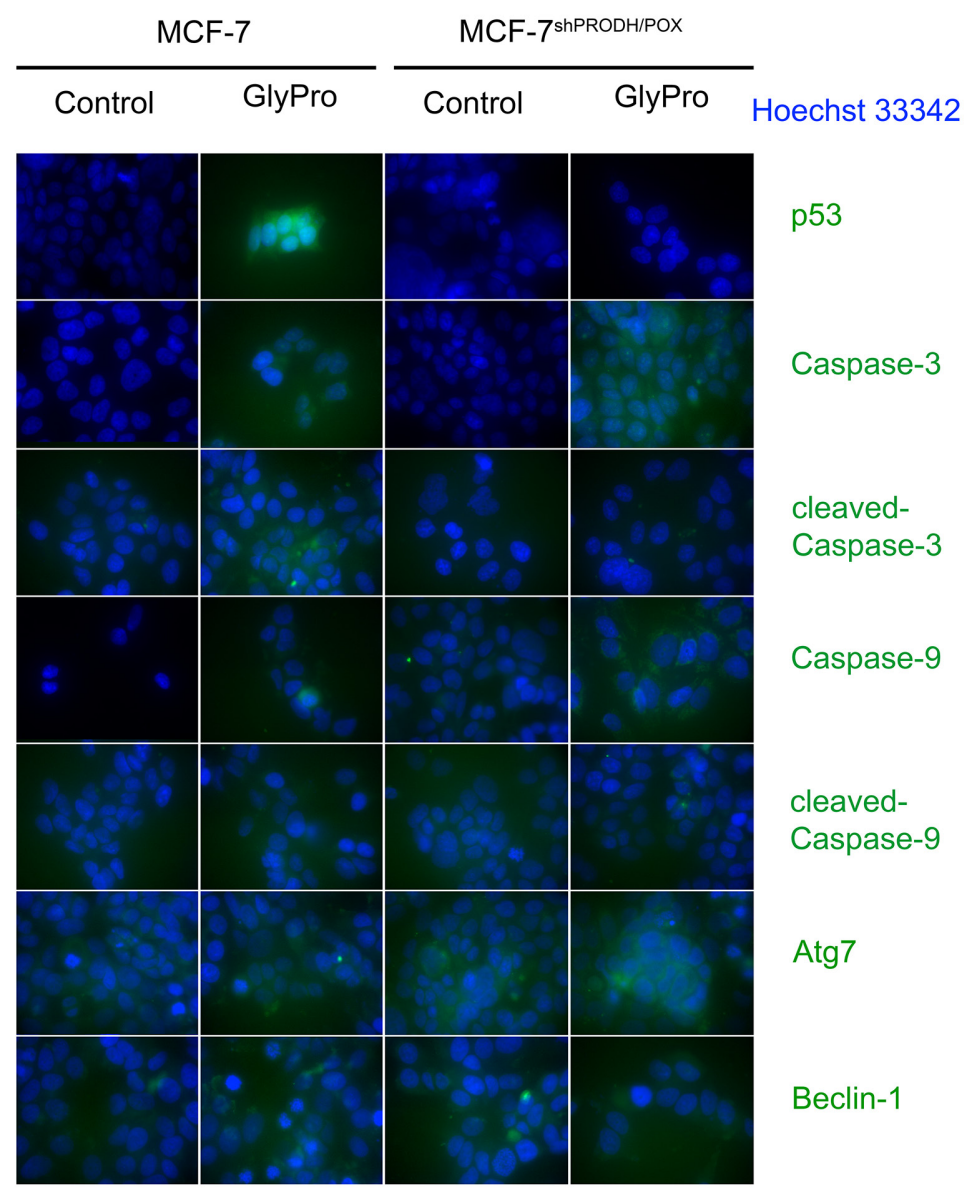
Confocal microscopy bio-imaging of p53, cleaved and un-cleaved caspase-3 and caspase-9, Atg7 and Beclin-1 in MCF-7 and MCF-7^shPRODH/POX^ cells treated with glycyl-proline (GlyPro)

The effect of PRODH/POX silencing and GlyPro on expression of p53, cleaved and un-cleaved Caspase-3, Caspase-9, Atg7 and Beclin-1 in MCF-7 and MCF-7^shPRODH/POX^ cells was confirmed by immunofluorescence bioimaging (Figure 3).

## DISCUSSION

We provided evidence that proline availability for PRODH/POX-dependent degradation augmented pro-survival phenotype in PRODH/POX silenced MCF-7cells. Proline concentration in cytoplasm depends on prolidase activity (enzyme releasing proline from imidodipeptides, as e.g. GlyPro) and collagen biosynthesis (process that utilizes proline for the protein biosynthesis). Therefore, the role of both processes (prolidase activity and collagen biosynthesis) in regulation of substrate availability (proline) for PRODH/POX-degradation was studied. In this manuscript we suggest that in PRODH/POX knock-down MCF-7 cells (expressing about 50% PRODH/POX of control MCF-7 cells), proline availability facilitate cell survival. It happens when proline concentration in cytoplasm increases as a result of GlyPro degradation by prolidase. Therefore, we suggest that proline accumulation induces PRODH/POX-dependent pro-survival pathways in PRODH/POX silenced MCF-7 human breast cancer cells.

We suggest that the switching from survival to apoptotic mode may depend on PRODH/POX activity and proline availability for PRODH/POX. Recent data showed down-regulation of PRODH/POX expression in various type of cancer [37]. Proline, the substrate for PRODH/POX enzyme is considered as a stress molecule, and can be utilized from cytoplasm by incorporation into collagen [38]. Therefore, collagen biosynthesis can be considered as a “sink” for proline. The functional significance of this process is removal of reducing potential of proline. Removal of proline by up-regulation of collagen biosynthesis may however promote tissue cirrhosis, which frequently is accompanied by prolonged inflammation [39, 40]. Alternatively, mitochondrial conversion of proline into P5C promotes ROS-dependent apoptosis [41]. On the other hand, free cytoplasmic proline might stabilize transcriptional activity of HIF-1 α. This reaction is considered as inflammatory and pro-survival process since HIF-1 α induces expression of COX-2, VEGF, TNF-α, IL-1, NF-κB and several other genes of great importance in inflammatory response [15]. Therefore, proline availability and specific pathway of proline metabolism may represent regulatory mechanism for cell survival/apoptosis.

Most of free proline is produced from imidodipeptides by prolidase. Critical factor in regulation of intracellular proline concentration is prolidase activity. The enzyme is regulated by signals from β1integrin receptor [16] and phosphorylation of the enzyme on threonine and tyrosine residues [42]. However, other mechanisms that contribute to generation as well as utilization of intracellular proline are also important.

In this study, we found that in MCF-7^shPRODH/POX^ cells, prolidase substrate (GlyPro) inhibited collagen biosynthesis while in MCF-7 cells, it contributed to increase in collagen biosynthesis, compared to control MCF-7 cells. The rate of collagen biosynthesis was correlated with expression of NF-κB - the inhibitor of collagen gene expression [43, 44]. In MCF-7^shPRODH/POX^ cells expression of NF-κB is higher than in MCF-7 cells. Moreover, it seems that increase in proline level (due to GlyPro cleavage by prolidase) and inhibition of proline utilization into P5C impairs regeneration of oxidizing potential, resulting in suppression of collagen biosynthesis [45, 46]. The same mechanism may apply to the cell proliferation. We also considered PARP, which is involved in DNA repair in response to stress, but there was no expression of active form of this protein in both cell lines. The mechanism of inhibition of cell proliferation in MCF-7^shPRODH/POX^ cells is likely to be associated with modulation of cell signalling pathways and cell cycle regulatory processes, because it was found modulation of EGFR, COX-2 and MAPK signalling and Wnt/β-catenin pathway by PRODH/POX [27, 29, 30, 37]. Therefore, intriguing is observation of increased prolidase activity in MCF-7^shPRODH/POX^ cells suggesting the functional link between prolidase and PRODH/POX. We considered these enzymes as a molecular inter-face that can switch on and off survival or apoptotic mode. We found that up-regulation of proline concentration in cytoplasm contributed to induction of apoptosis in MCF-7 cells, while in MCF-7^shPRODH/POX^ cells the process was inhibited, inducing survival mode. In fact, in the presence of proline, overexpression of PRODH/POX causes cytochrome c release from mitochondria to cytosol and activation of caspase-9 and caspase-3 [9, 33]. Therefore, PRODH/POX may play dual role, but the mechanism that switches PRODH/POX from cell growth inhibiting to growth supporting factor is still unknown. Some explanation is provided by other studies. During conversion of proline into P5C, electrons are transported to electron transport chain generating ATP or they directly reduce oxygen, producing reactive oxygen species (ROS). In situation, when the glucose level is low, the consequence of activation of PRODH/POX is production of ATP for energy supply and survival [1, 5, 6]. Otherwise, direct reduction of oxygen by electrons generate ROS that induce apoptosis [7–10].

We considered another factors, that may play important role in the switching mechanism of apoptosis/autophagy, namely prolidase and HIF-1 α.

Some studies documented that in the presence of prolidase substrate, GlyPro, overexpression of prolidase contributed to increase in HIF-1 α expression and HIF-1 α transcriptional activity. The prolidase-dependent differences in HIF-1 α expression was also shown in two breast cancer cell lines, characterized by different expression of prolidase. These findings show that GlyPro degradation by prolidase play an important role in survival pathways [15].

Although PRODH/POX catalyses proline conversion to P5C, in the next steps it is converted into glutamate and α-ketoglutaric acid, which inhibits transcriptional activity of HIF-1. The α-ketoglutaric acid is co-substrate of prolyl hydroxylase domain (PHD). Increase in PHD activity contributes to increase in HIF-1α degradation and consequently to decrease in HIF-1-dependent gene expressions [32, 47]. It was suggested that PRODH/POX-dependent down-regulation of HIF-1 signalling may affect cell cancer invasion, tumour growth and angiogenesis. Protein p53 is considered as the most potent activator of PRODH/POX [31, 48]. The evidence for transcriptional regulation of PRODH/POX by p53 was presented by Maxwell and Kochevar. They found that PRODH/POX promoter contained a p53-response element [49]. Furthermore, p53 is activator of PUMA protein, which promotes apoptosis by binding to and antagonizing anti-apoptotic Bcl-2 family members. In our study, we found that in both GlyPro treated and untreated MCF-7 cells the expression of PUMA was higher compared to MCF-7^shPRODH/POX^ cells.

Although PRODH/POX was found as tumour suppressor, in certain environmental conditions it may act as a pro-survival factor [1, 5, 30, 33–36]. In some stress situations (metabolic, oxygen and glucose deficiency, inflammation, genotoxicity) PRODH/POX activation support tumour growth. Hypoxia and glucose deficiency may evoke additive effects on PRODH/POX expression, directly through AMPK (AMP-activated protein kinase) activation and mTOR (the mammalian target of rapamycin) pathway which depends on nutrient’s availability [33, 50]. Its knockdown by rapamycin also up-regulated PRODH/POX [30, 35, 50].

Our data show that in MCF-7^shPRODH/POX^ cells mTOR is up-regulated and the process is supported by prolidase substrate (GlyPro). Therefore, we considered proline as a stress molecule, that when accumulated in cytoplasm and subsequently in mitochondria, facilitates PRODH/POX-dependent apoptosis. In this study we found that PRODH/POX silencing strongly increased expression of Atg7 and Beclin-1, as autophagy markers, as well as iNOS, NF-κB, AMPK α, COX-2 in GlyPro treated and untreated cells.

PRODH/POX-dependent autophagy/apoptosis is dependent on metabolic context. For instance, glucose depletion enhances PRODH/POX expression through AMPK to promote cancer cell survival through autophagy. Autophagy plays an essential role in the maintenance of cellular energy for cell survival in stress conditions. Activation of AMPK are among the major regulators of autophagy [51]. It has been reported that the early stage of autophagy in cancer cells is regulated by several ‘Atg’ (Autophagy-related genes) and proteins which have been implicated in autophagosome formation [52]. Atg7 and Beclin-1 are required to recruit proteins to the autophagosomal membrane and to form the autophagic vacuole presented to citric acid cycle for energy generation [53, 54]. Therefore, it seems that down regulation of PRODH/POX may facilitate autophagy for cancer cell survival, while up-regulation of this enzyme may create condition for apoptosis.

Of special interest is that in MCF-7 cells GlyPro derived proline induced apoptosis, as detected by up-regulation of expression of active caspase-3, active caspase-9, PUMA, while in MCF-7^shPRODH/POX^ cells the opposite process was observed with induction of survival mode, as detected by down-regulation of caspases and up-regulation of HIF-1α, NF-κB, COX-2, AMPK α, Atg7 and Beclin-1 expression.

Summarizing, we demonstrated that MCF-7^shPRODH/POX^ cells express pro-survival phenotype and proline (derived from GlyPro) supports the process. In contrast, in MCF-7 cells, proline activated pro-apoptotic signalling pathways.

## MATERIALS AND METHODS

### Cell lines and culture

Breast cancer cell line MCF-7 was obtained from ATCC (HTB-22, ATCC, Manassas, VA, USA). The MCF-7 and MCF-7^shPRODH/POX^ cells were maintained in DMEM and 5% fetal bovine serum (FBS, Gibco, Thermo Fisher Scientific, Waltham, Massachusetts, USA), 50 IU/ml penicillin (Gibco), and 50 μg/ml streptomycin (Gibco) at 37 °C in a humidified atmosphere in the presence of 5% CO_2_. Description of the preparation of MCF-7^shPRODH/POX^ cell line was included in supplementary data (Supplemental Experimental Procedures, Supplementary Figure 1). In the experimental conditions 80% of confluent MCF-7 and MCF-7^shPRODH/POX^ cells were cultured in glutamine-free DMEM (Gibco) (in order to avoid proline generation from glutamine) and treated for 24 h with substrate for prolidase, GlyPro (17,22 μg/ml).

### Western-immunoblot analysis

Cell lysates of MCF-7^shPRODH/POX^ and control MCF-7 cells (treated and untreated with GlyPro) were harvested and subjected to SDS-PAGE in 10% polyacrylamide gel electrophoresis [1h, 125 V, room temperature (RT)]. The protein was transferred to 0.2 μm pore-sized nitrocellulose (wet transfer, 1 h, 100 mA, RT). After the transfer, membranes were blocked with 5% non-fat dry milk in TBS-T (20 mmol/l Tris–HCl, 150 mmol/l NaCl, 0.05% Tween 20, pH 7.4) and incubated with goat anti-PRODH/POX antibodies (Everest Biotech, Upper Heyford, UK), rabbit anti-COX-2 (B&D), mouse anti-HIF-1α (Becton, Dickinson and Company (B&D), New Jersey, USA), mouse anti-NF-κB (B&D), mouse anti-iNOS (B&D), rabbit anti-caspase-3 (Cell Signaling (CS), Danvers, USA), rabbit anti-cleaved-caspase-3 (CS), rabbit anti-caspase-9 (CS), mouse anti-cleaved- caspase-9 (B&D), ), rabbit anti-PUMA (CS), mouse wild-type anti-p53 (B&D), rabbit anti-PARP (CS), rabbit anti-cleaved-PARP (CS), rabbit anti-Atg5 (CS), rabbit anti-Atg7 (CS), rabbit anti-Beclin-1 (CS), rabbit anti-mTOR (CS), rabbit anti-AMPKα (CS), mouse anti-AMPKβ (B&D), mouse anti-β-actin (Sigma-Aldrich, Saint Louis, Missouri, USA) diluted 1:1000 in blocking buffer. Then membranes were washed in TBS with 0.05% Tween (TBST) 3 x 15 min and incubated with respective HRP-linked secondary antibody at concentration 1:7500 (Sigma-Aldrich) for 60 min at RT with gentle agitation. After washing in TBS-T (5 × 5 min) membranes were incubated with Amersham ECL Western Blotting Detection Reagent, (GE Healthcare Life Sciences, Little Chalfont, Buckinghamshire, UK). Pictures were taken using BioSpectrum Imaging System UVP (Ultra-Violet Products Ltd, Cambridge, UK).

### Cell viability assay

The cell viability was determined using Nucleo Counter NC-3000 (ChemoMetec, Copenhagen, Denmark). Prior the experiment MCF-7 and MCF-7^shPRODH/POX^ cells were cultured in six-well plates at 1 × 10^5^ cells/well with 2 ml of growth medium. After 24 h incubation of the cells in glutamine-free DMEM with or without GlyPro, medium was discarded and the cells were rinsed three times with phosphate buffered saline (PBS). Then the cells were harvested, washed and stained with VitaBright-48 (VB-48) (ChemoMetec), acridine orange (AO) (ChemoMetec), propidium iodide (PI) (ChemoMetec) and analyzed using NC-3000 cell counter.

### DNA biosynthesis assay

Proliferation of MCF-7 and MCF-7^shPRODH/POX^ cells was measured by [methyl-^3^H]thymidine (Hartman Analytic GmbH, Braunschweig, Germany) incorporation into DNA. Prior the experiment MCF-7 and MCF-7^shPRODH/POX^ cells were cultured in 24-well plate at 1 × 10^4^ cells/well with 1 ml of growth medium. After 48 h the cells were incubated in glutamine-free DMEM (Gibco) with or without GlyPro for 24 h and next with 0.5 μCi/ml of [methyl-^3^H]thymidine for 4 h. PBS-rinsed cells were solubilized with 1 ml of 0.1 mol/l sodium hydroxide containing 1% SDS and 5 ml of scintillation fluid Ultima Gold XR (Perkin Elmer, Waltham, USA). Incorporation of the tracer into DNA was measured by LiquidScintillation Analyzer Tri-Carb 2810 TR (Perkin Elmer) and calculated using QuantoSmart TM software (Perkin Elmer).

### Collagen biosynthesis

Incorporation of radioactive precursor into proteins was measured after the labeling of 80% confluent cells cultured in glutamine-free DMEM medium with 5[^3^H]-proline (5 μCi/ml) and with or without GlyPro for 24 h. Incorporation of tracer into collagen was determined by digesting proteins with purified Clostridium histolyticum collagenase, according to the method of Peterkofsky et al. [55]. Results are shown as combined values for cell plus medium fractions.

### Determination of prolidase activity

The activity of prolidase was determined according to the method of Myara et al. [56]. Protein concentration was measured by the method of Lowry et al. [57]. Enzyme activity was reported as nanomoles of proline released from synthetic substrate (GlyPro), during 1 min per milligram of supernatant protein of cell homogenate.

### Immunofluorescence microscopy

Cells grown on a coverslip were fixed with 3,7% paraformaldehyde and permeabilized with 0.01% Triton. After blocking with 3% foetal bovine serum, cells were incubated with primary antibodies (p53, caspase-3, cleaved-caspase-3 caspase-9, cleaved-caspase-9, atg7, beclin-1) at dilutions 1:500, and subsequently with FITC Fluor-conjugated secondary antibody (Becton, Dickinson and Company, USA). Sample were visualized with a confocal laser scanning microscope (BD Pathway 855 Bioimager) using AttoVision software.

### Concentration of proline

Samples were analyzed by an HPLC system (1260 Infinity series, Agilent Technologies, Waldbronn, Germany) consisting of a degasser, binary pump, and thermostated autosampler maintained at 4°C connected to an Agilent Technologies QTOF (6530) mass spectrometry detector. Electrospray ionization (ESI) was used as an ion source in positive ionisation mode. Samples (2 μL) were injected onto a HILIC column (Luna HILIC, 100x2.0mm; 3um; Phenomenex) thermostated at 40 °C. The system was operated in positive and negative mode at flow rate 1 mL/min with solvent A - water with 10mM ammonium formate (70221, Sigma-Aldrich) and solvent B-acetonitrile/water (9:1, v:v) with 10mM ammonium formate. Mobile phase was 100% B during 1.5min in isocratic mode. The gradient started in 1.5 min from 100% B to 70% B in 5.5min, then 40% B in 6.0min, maintained 40% B during 1 min and returned to starting conditions in 0.5 min, keeping the re-equilibration until 10 min. The detector operated in full scan mode from 50 to 1000 m/z with a scan rate of 1 scan per second. Accurate mass measurements were obtained by online mass correction to reference masses delivered continuously during analyses. Reference masses at m/z 121.0509 (protonated purine) and m/z 922.0098 [protonated hexakis (1H,1H,3Htetrafluoropropoxy) phosphazine or HP-921]. The capillary voltage was set to 3000V, the gas temperature was 330°C, the nebulizer gas flow rate was 10,5 L/min. MS TOF parameters were as follows: fragmentor was set to 140V, skimmer 65 V.

### Statistical analysis

In all experiments, the mean values for six assays ± standard deviations (S.D.) were calculated. The results were submitted to the statistical analysis using the Student’s “*t* ”-test and two-way ANOVA, accepting ^*^P<0.05, ^**^P<0.01 and ^***^P<0.001.

## SUPPLEMENTARY MATERIALS FIGURES AND TABLE


