# Supplementary material for: Proline oxidase silencing induces proline-dependent pro-survival pathways in MCF-7 cells
Source: Oncotarget. 2018 Feb 9;9(17):13748–57. doi: 10.18632/oncotarget.24466 (PMC5862612; doi:10.18632/oncotarget.24466)
Supplement: Supplementary file 1 [file oncotarget-09-13748-s001.pdf]

Proline oxidase silencing induces proline-dependent pro-survival pathways in MCF-7 cells

SUPPLEMENTARY MATERIALS

SUPPLEMENTARY DATA

Proline Oxidase silencing induces proline-dependent pro-survival pathways in MCF-7 cells.

RESULTS

Efficacy of shRNA-based *PRODH/POX* knock-down in MCF-7 cells

Designed shRNA constructs targeted exons 3 and 2 (PRODH shRNA1), exons 14 and 13 (PRODH shRNA2) and exons 15 and 14 (PRODH shRNA3) of transcription variant 1 and 2, respectively. Western blot protein analysis showed that the PRODH shRNA 2 construct was the most effective one in silencing PRODH/POX, decreasing its expression to about 35% of controlMCF-7 cells (Supplementary Figure 1). This cell clone was named MCF-7$^{shPRODH/POX}$ and selected for further *in vitro* analysis presented in the main manuscript.

Cell viability, DNA and collagen biosynthesis, prolidase activity and intracellular proline concentration in MCF-7 PRODH/POX knock-down cells (MCF-7$^{shPRODH/POX}$) versus MCF-7 cells

The studies were performed on 3 clones of PRODH/POX silenced MCF-7 cells and MCF-7 control cells treated with GlyPro (glicyl-proline) as a substrate for prolidase in order to increase cytoplasmic level of proline (Supplementary Figure 2). There was no significant difference in cell viability between studied clones of MCF-7 cells, the cells transfected with empty vector and MCF-7 control cells either treated or untreated with GlyPro (Supplementary Figure 2A). However, in clone 2 and clone 3 of MCF-7 cells, DNA biosynthesis was significantly decreased compared to MCF-7 control cells (Figure 2B). Although, GlyPro further inhibited the DNA biosynthesis in cells of clone 1 and clone 2, there was not significant effect of GlyPro on the process in clone 3 MCF-7 cells.

Collagen biosynthesis was decreased in clone 2 MCF-7 cells (but not in clone 1) and GlyPro inhibited the process in clone 1 and clone 2 (Supplementary Figure 2C). However, collagen biosynthesis was not affected in clone 3 MCF-7 cells in both GlyPro treated and untreated cells.

Prolidase activity and intracellular proline concentration were significantly increased in clone 2 MCF-7 cells in both GlyPro treated and untreated cells (Supplementary Figure 2D, Supplementary Figure 2E).

The data suggest that clone 2 MCF-7 cells represent the most characteristic phenotype to study the role of deregulation of proline generation/utilization processes on apoptosis/autophagy. Therefore, we used clone 2 MCF-7 cells for further studies.

Effect of PRODH/POX silencing and GlyPro on pro-apoptotic and pro-survival pathways in MCF-7 cells

The data for quantitation of protein expression are presented on Figure 3.

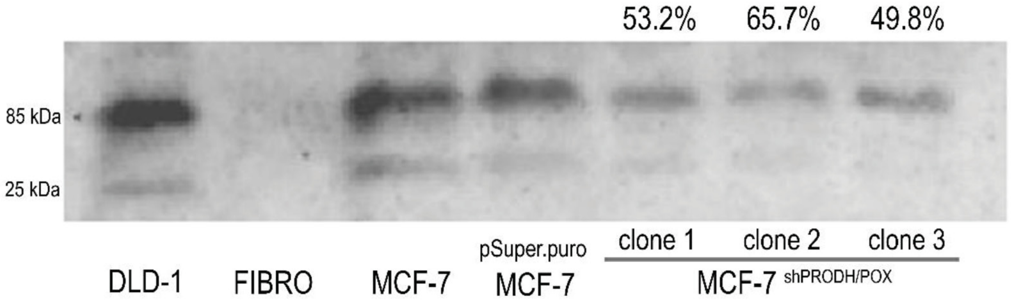

Supplementary Figure 1: Expression of PRODH/POX in MCF-7 and MCF-7$^{shPRODH/POX}$ cells. Transfection of the MCF-7cells with different PRODH/POX shRNA constructs (clone 1-3) were done. Fibroblasts were used as a negative control and colorectal cancer cells DLD-1, as positive control for the expression of PRODH/POX.

MATERIALS AND METHODS

shRNA-based *PRODH* knock-down

To knock-down expression of *PRODH*, 3 pairs of short herpins complement single-stranded DNA oligonucleotides were designed using AsiDesigner online tool (http://sysbio.kribb.re.kr:8080/AsiDesigner/menuDesigner.jsf) (Supplementary Table 1). HPLC purified oligonucleotides were ordered from Genomed (Warsaw, Poland). Annealed double-stranded oligonucleotides were cloned into pSuper.puro expression vector (OligoEngine, Seattle, WA, USA) via BglII and HindIII restriction sites using T4 DNA ligase (Thermo Fisher Scientific, Waltham, Massachusetts, USA). Positive colonies of heat-shock transformed NEB 10-beta competent *E. coli* bacteria (Bio Labs, Ipswich, United Kingdom), were verified by PCR, restriction enzyme BglII and HindIII digestion (Thermo Fisher Scientific), and gel electrophoresis. Amplified plasmid DNA was extracted and purified by NucleoSpin® Plasmid (Macherey-Nagel, Düren, Germany). Oligonucleotide sequences (shRNA oligos) used in the study, PRODH1 (NM_016335.4), PRODH2 (NM_001195226.1) are shown in Supplementary Table 1. The sequences used to silence PRODH/POX expression were subject for patent application (patent application number: P.421954).

MCF-7 cells were cultured in 6-well plate (1x10^5 cells per well in 2 ml culture media). Twenty-four hours later, cells were transfected with three different shRNA expressing vectors or shRNA non-targeting control using Lipofectamine™ 2000 (Invitrogen, Thermo Fisher Scientific) in OptiMem media (Gibco) according to the manufacturer's protocol. *PRODH* shRNA-expressing cells were selected using puromycin antibiotic (Invitrogen) with previously optimized concentration of 1 µg/ml. The MCF-

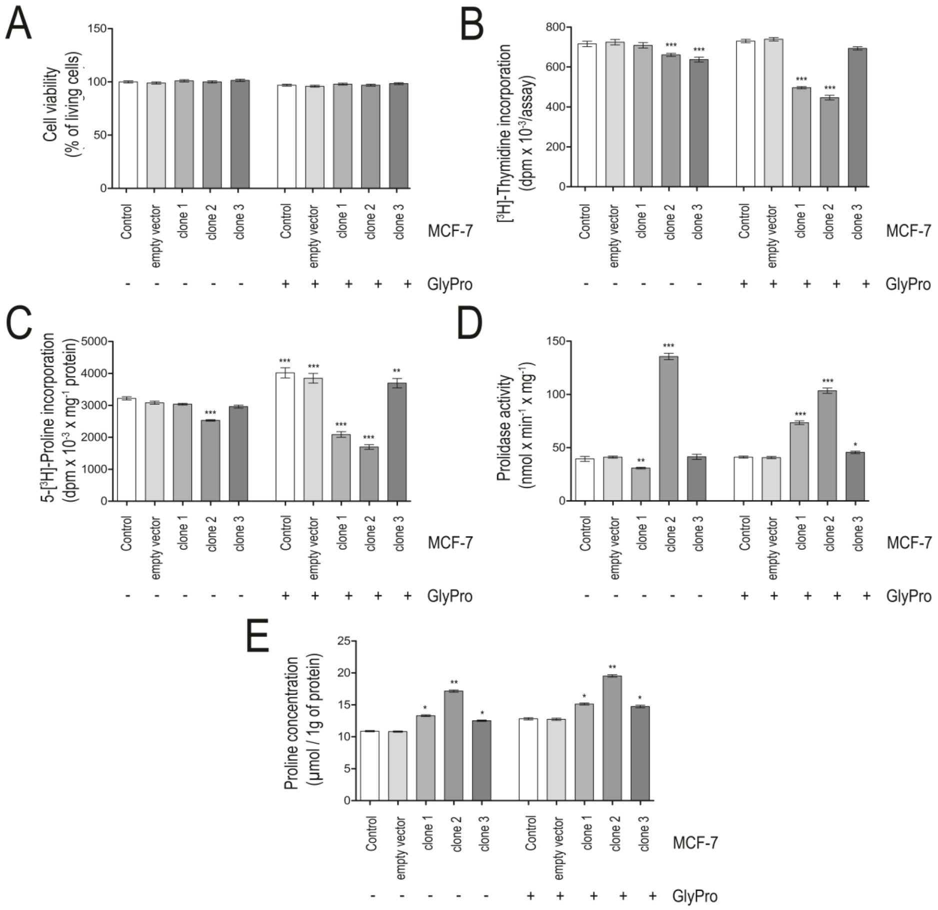

Supplementary Figure 2: Effect of PRODH/POX silencing and GlyPro on cell viability **(A)**, DNA biosynthesis **(B)**, collagen biosynthesis **(C)**, prolidase activity **(D)** and intracellular proline concentration **(E)** in MCF-7 and clones of PRODH/POX silenced MCF-7 cells. The mean values ± SEM from 3 experiments done in duplicates are presented. Asterisks indicate differences between control MCF-7 cells and clones of PRODH/POX silenced MCF-7 cells at *P<0.05, **P<0.01 and ***P<0.001.

Supplementary Table 1: Oligonucleotide sequences (shRNA oligos) used in the study, PRODH1 (NM_016335.4), PRODH2 (NM_001195226.1)

Name		Sequences (5’- 3’)	Target
PRODH shRNA 2	F:	**gatccccCTAGGACAGAGGCTATTCAACttcaagagaGTTGAATAGCCTCTGTCCTAGttttta**	*PRODH tv. 1* 502-522
	R:	**agcttaaaaaCTAGGACAGAGGCTATTCAACtctcttgaaGTTGAATAGCCTCTGTCCTAGggg**	*PRODH tv. 2* 87-107
PRODH shRNA 1	F:	**gatccccGCATGTGTGACCAGATCAGCTttcaagagaAGCTGATCTGGTCACACATGCttttta**	*PRODH tv. 1* 1790-1810
	R:	**agcttaaaaaGCATGTGTGACCAGATCAGCTtctcttgaaAGCTGATCTGGTCACACATGCggg**	*PRODH tv. 2* 1374-1394
PRODH shRNA 3	F:	**gatccccGTGTACAAGTACGTGCCCTATttcaagagaATAGGGCACGTACTTGTACACttttta**	*PRODH tv. 1* 1837-1857
	R:	agcttaaaaa**GTGTACAAGTACGTGCCCTAT**tctcttgaaa**TAGGGCACGTACTTGTACAC**ggg	*PRODH tv. 2* 1422-1442

tv - transcript variant.
Oligonucleotide sequences used in the study, PRODH1 (NM_016335.4) sequences in the shRNA oligos are in bold.

7 stable transfected cells were generated from selecting the single puromycin-resistant cell and were further characterized and used in this study.

Western-immunoblot analysis

Cell lysates of MCF-7shPRODH/POX and control MCF-7 cells (treated and untreated with GlyPro) were harvested and subjected to SDS-PAGE in 10% polyacrylamide gel electrophoresis [1h, 125 V, room temperature (RT)]. The protein was transferred to 0.2 µm pore-sized nitrocellulose (wet transfer, 1 h, 100 mA, RT). After the transfer, membranes were blocked with 5% non-fat dry milk in TBS-T (20 mmol/l Tris–HCl, 150 mmol/l NaCl, 0.05% Tween 20, pH 7.4) and incubated with goat anti-PRODH/POX antibodies (Everest Biotech, Upper Heyford, UK), rabbit anti-COX-2 (B&D), mouse anti-HIF-1α (Becton, Dickinson and Company (B&D), New Jersey, USA), mouse anti-NF-κB (B&D), mouse anti-iNOS (B&D), rabbit anti-caspase-3 (Cell Signaling (CS), Danvers, USA), rabbit anti-cleaved-caspase-3 (CS), rabbit anti-caspase-9 (CS), mouse anti-cleaved-caspase-9 (B&D),), rabbit anti-PUMA (CS), mouse (wild-type) anti-p53 (B&D), rabbit anti-PARP (CS), rabbit anti-cleaved-PARP (CS), rabbit anti-Atg5 (CS), rabbit anti-Atg7 (CS), rabbit anti-Beclin-1 (CS), rabbit anti-mTOR (CS), rabbit anti-AMPKα (CS), mouse anti-AMPKβ (B&D), mouse anti-β-actin (Sigma-Aldrich, Saint Louis, Missouri, USA) diluted 1:1000 in blocking buffer. Then membranes were washed in TBS with 0.05% Tween (TBST) 3 x 15 min and incubated with respective HRP-linked secondary antibody at concentration 1:7500 (Sigma-Aldrich) for 60 min at RT with gentle agitation. After washing in TBS-T (5 × 5 min) membranes were incubated with Amersham ECL Western Blotting Detection Reagent, (GE Healthcare Life Sciences, Little Chalfont, Buckinghamshire, UK). Pictures were taken using BioSpectrum Imaging System UVP (Ultra-Violet Products Ltd, Cambridge, UK). Densitometry of western blot bands was analyzed using ImageJ software.

Cell lines and culture

Breast cancer cell line MCF-7 was obtained from ATCC (HTB-22, ATCC, Manassas, VA, USA). The MCF-7 and MCF-$7^{shPRODH/POX}$ cells were maintained in DMEM and 5% fetal bovine serum (FBS, Gibco, Thermo Fisher Scientific, Waltham, Massachusetts, USA), 50 IU/ml penicillin (Gibco), and 50 µg/ml streptomycin (Gibco) at 37 °C in a humidified atmosphere in the presence of 5% CO_2. In the experimental conditions 80% of confluent MCF-7 and MCF-$7^{shPRODH/POX}$ cells were cultured in glutamine-free DMEM (Gibco) (in order to avoid proline generation for glutamine) and treated for 24 h with substrate for prolidase GlyPro (17,22 µg/ml).

Cell viability assay

The cell viability was determined using Nucleo Counter NC-3000 (ChemoMetec, Copenhagen, Denmark). Prior the experiment MCF-7 and MCF-$7^{shPRODH/POX}$ cells were cultured in six-well plates at 1×10^5 cells/well with 2 ml of growth medium. After 24 h incubation of the cells in glutamine-free DMEM with or without GlyPro, medium was discarded and the cells were rinsed three times with phosphate buffered saline (PBS). Then the cells were harvested, washed and stained with VitaBright-48 (VB-48) (ChemoMetec), acridine orange (AO) (ChemoMetec), propidium iodide (PI) (ChemoMetec) and analyzed using NC-3000 cell counter.

DNA biosynthesis assay

Proliferation of MCF-7 and MCF-$7^{shPRODH/POX}$ cells was measured by [methyl-^{3}H]thymidine (Hartman Analytic GmbH, Braunschweig, Germany) incorporation into DNA. Prior the experiment MCF-7 and MCF-$7^{shPRODH/POX}$ cells were cultured in

24-well plate at 1 × 10^4 cells/well with 1 ml of growth medium. After 48 h the cells were incubated in glutamine-free DMEM (Gibco) with or without GlyPro for 24 h and next with 0.5 µCi/ml of [methyl-^{3}H]thymidine for 4 h. PBS-rinsed cells were solubilized with 1 ml of 0.1 mol/l sodium hydroxide containing 1% SDS and 5 ml of scintillation fluid Ultima Gold XR (Perkin Elmer, Waltham, USA). Incorporation of the tracer into DNA was measured by LiquidScintillation Analyzer Tri-Carb 2810 TR (Perkin Elmer) and calculated using QuantoSmart TM software (Perkin Elmer).

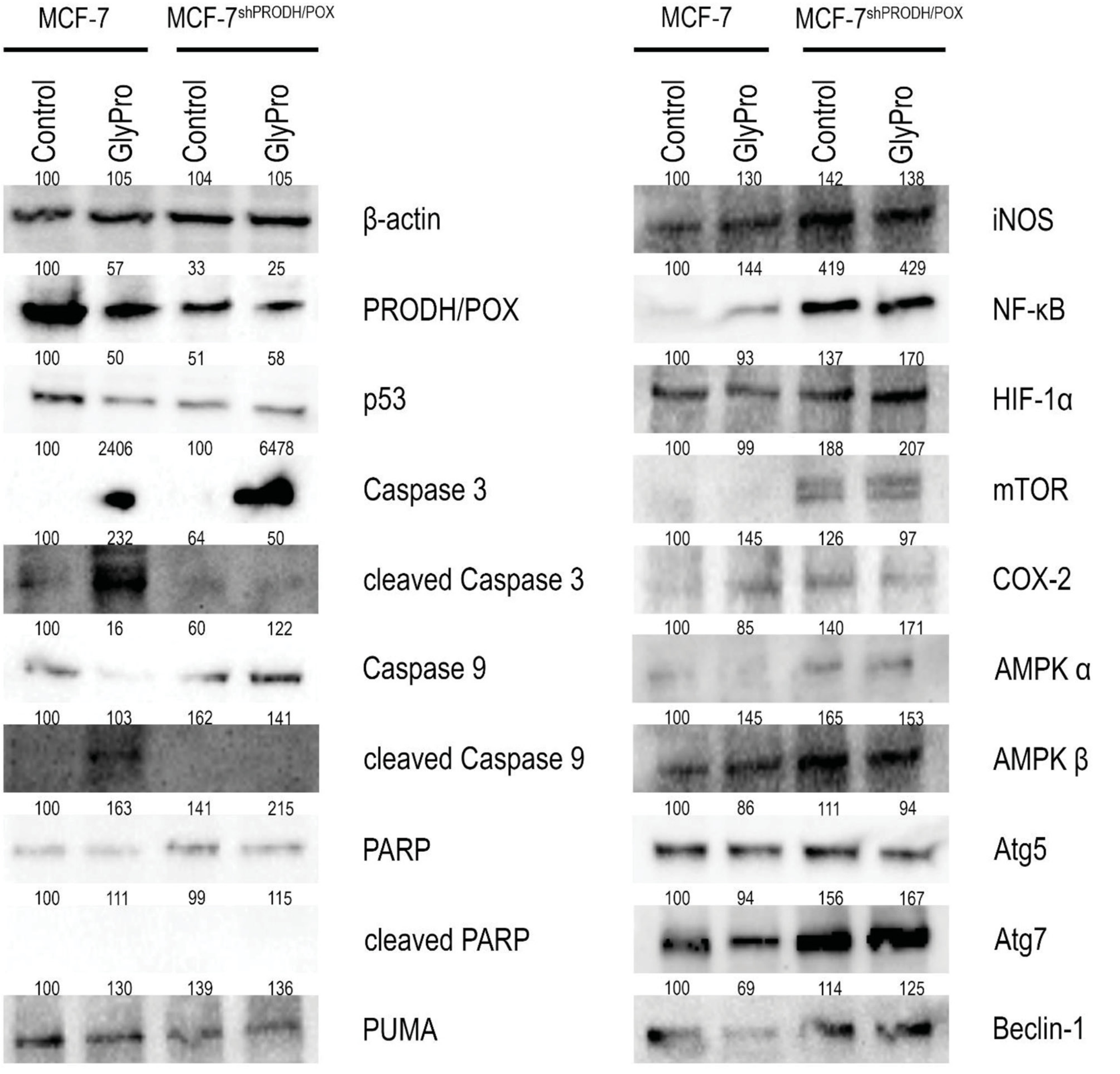

Supplementary Figure 3: Effect of PRODH/POX silencing and GlyPro on pro-apoptotic and pro-autophagy signaling in MCF-7 cells. Western blot analysis for PRODH/POX, p53, Caspase 3, cleaved-Caspase 3, Caspase-9, cleaved-Caspase 9, PARP, cleaved-PARP, PUMA, iNOS, NF-κB, HIF-1α, mTOR, COX-2, AMPKα, AMPKβ, Atg5, Atg7 and Beclin-1 in MCF-7 and MCF-7$^{shPRODH/POX}$ cells and the cells treated with GlyPro. Representative blots obtained from 3 experiments done in duplicates are presented. Samples used for electrophoresis consisted of 20 µg protein of cells homogenate extracts. The intensity of the bands staining was quantified by densitometry analysis. The mean values are presented.

Collagen biosynthesis

Incorporation of radioactive precursor into proteins was measured after the labeling of 80% confluent cells cultured in glutamine-free DMEM medium with 5[^{3}H]-proline (5 μCi/ml) and with GlyPro for 24 h. Incorporation of tracer into collagen was determined by digesting proteins with purified Clostridium histolyticum collagenase, according to the method of Peterkofsky et al. (31). Results are shown as combined values for cell plus medium fractions.

Determination of prolidase activity

The activity of prolidase was determined according to the method of Myara et al. (32). Protein concentration was measured by the method of Lowry et al. (33). Enzyme activity was reported as nanomoles of proline released from synthetic substrate (GlyPro), during 1 min per milligram of supernatant protein of cell homogenate.

Concentration of proline

Samples were analyzed by an HPLC system (1260 Infinity series, Agilent Technologies, Waldbronn, Germany) consisting of a degasser, binary pump, and thermostated autosampler maintained at 4°C connected to an Agilent Technologies QTOF (6530) mass spectrometry detector. Electrospray ionization (ESI) was used as an ion source in positive ionisation mode. Samples (2 μL) were injected onto a HILIC column (Luna HILIC, 100x2.0mm; 3um; Phenomenex) thermostated at 40 °C. The system was operated in positive and negative mode at flow rate 1 mL/min with solvent A - water with 10mM ammonium formate (70221, Sigma-Aldrich) and solvent B – acetonitrile/ water (9:1, v:v) with 10mM ammonium formate. Mobile phase was 100% B during 1.5min in isocratic mode. The gradient started in 1.5 min from 100% B to 70% B in 5.5min, then 40% B in 6.0min, maintained 40% B during 1 min and returned to starting conditions in 0.5 min, keeping the re-equilibration until 10 min. The detector operated in full scan mode from 50 to 1000 m/z with a scan rate of 1 scan per second. Accurate mass measurements were obtained by online mass correction to reference masses delivered continuously during analyses. Reference masses at m/z 121.0509 (protonated purine) and m/z 922.0098 [protonated hexakis (1H,1H,3Htetrafluoropropoxy) phosphazine or HP-921]. The capillary voltage was set to 3000V, the gas temperature was 330°C, the nebulizer gas flow rate was 10,5 L/min. MS TOF parameters were as follows: fragmentor was set to 140V, skimmer 65 V.

Statistical analysis

In all experiments, the mean values for six assays ± standard deviations (S.D.) were calculated. The results were submitted to the statistical analysis using the Student's "t"-test and Two-way ANOVA, accepting $^{*}P<0.05$, $^{**}P<0.01$ and $^{***}P<0.001$ as significant.
